# NVP-AUY922 alleviates radiation-induced lung injury via inhibition of autophagy-dependent ferroptosis

**DOI:** 10.1038/s41420-022-00887-9

**Published:** 2022-02-26

**Authors:** Li Li, Dongming Wu, Shihua Deng, Jin Li, Feng Zhang, Ye Zou, Ting Zhang, Ying Xu

**Affiliations:** 1 School of Clinical Medicine, Chengdu Medical College, Chengdu, Sichuan 610500 P.R. China; 2 The First Affiliated Hospital of Chengdu Medical College, Chengdu, Sichuan 610500 P.R. China

**Keywords:** Diseases, Drug discovery

## Abstract

Radiation-induced lung injury (RILI) is a common complication of radiotherapy for which no effective interventions are available. NVP-AUY922, a resorcinylic isoxazole amide drug, exhibits anti-inflammatory, immunomodulatory, and therapeutic effects against various types of cancers. In this study, we explore the role and underlying mechanisms of NVP-AUY922 in the treatment of RILI. We established a model of BEAS-2B cell injury and a mouse model of RILI. Cell proliferation, death, gross weight, and survival rates of mice, and histological parameters were assessed. Additionally, inflammation-related indices and indicators related to ferroptosis were evaluated. Furthermore, immunofluorescence and co-immunoprecipitation were used to determine the interaction between GPX4, LAMP-2A, and HSC70. NVP-AUY922 significantly ameliorated radiation-induced lung tissue damage, inflammatory cell infiltration, proinflammatory cytokine release, and lung epithelial BEAS-2B cell damage. NVP-AUY922 markedly limited the activation of ferroptosis, which is involved in RILI. Mechanistically, NVP-AUY922 prevented chaperone-mediated autophagy of the GPX4 pathway in vitro and in vivo, and the autophagy inhibitor Baf-A1 significantly increased the level of GPX4 and alleviated lung inflammation. NVP-AUY922 can alleviate RILI by inhibiting chaperone-mediated lysosomal degradation of GPX4, demonstrating its potential as a novel protective agent against RILI.

## Introduction

Radiotherapy is a momentous and commonly used therapeutic modality for various tumors [1, 2]. Radiation-induced lung injury (RILI) occurs during the early stages of radiotherapy and is one of the independent risk factors for radiation-induced death [3]. Inflammation is a common and serious complication after radiation, and lung inflammation, in particular, is an early signal of lung damage [4]. The main clinical manifestations of RILI are inflammatory infiltration of alveolar interstitial material, progressive dyspnea, deterioration of lung function, and eventually respiratory failure [5]. Although some traditional radioprotective drugs have a protective effect on normal lung tissue, they weaken the sensitivity of tumor cells to radiation. These radioprotective drugs also greatly reduce the effect of tumor radiotherapy, restricting their application in chest radiotherapy for clinical tumor patients [6]. Therefore, a new radioprotective drug with significant curative effects, low toxicity, and side effects is necessary for the clinical treatment of RILI.

Cell death is a major early event in the occurrence of RILI, accompanied by inflammation caused by chemokines and injury-related molecules. Different forms of cell death patterns are involved in RILI [7]. Ferroptosis is characterized by the excessive accumulation of iron-dependent lipid peroxidation products, which leads to mitochondrial oxidative damage [8], and can be induced by down-regulation of system x_c_ − activity, inhibition of glutathione peroxidase 4 (*GPX4*), and elevated lipid reactive oxygen species (ROS) levels [9, 10]. Ferroptosis plays a crucial role in many diseases, such as neurological diseases [11], carcinogenesis [12], infections [13], and ischemia-reperfusion injury [14]. The relationship between ferroptosis and lung injury has recently been studied. Li et al. [15] found that ferroptosis plays a key role in RILI, and that liproxstatin-1, a ferroptosis inhibitor, alleviates RILI by downregulating transforming growth factor-β1 and activating the Nrf2 signaling pathway. Moreover, the authors showed that mitochondria in the RILI model had obvious changes in the characteristics of ferroptosis; the level of GPX4, a typical marker of ferroptosis, was reduced, and inflammation was significantly alleviated by a ferroptosis inhibitor [16]. Therefore, ferroptosis also plays a crucial role in acute RILI, but the underlying mechanism remains unclear.

Ferroptosis is an autophagy-dependent cell death mode [17, 18]. Chaperone-mediated autophagy (CMA) can typically be activated during starvation and stress stimulations, resulting in molecular degradation of cytoplasmic proteins [19]. A previous study showed that CMA activation is involved in GPX4 degradation; hence GPX4 is stabilized by inhibiting CMA and reducing ferroptosis [20]. Interestingly, autophagy affected the development and progression of diseases by forming a network with HSP90 in protein regulation [21], and CMA could recognize and degrade the customer protein of HSP90 using a special sequence.

NVP-AUY922 is a resorcinylic isoxazole amide that inhibits the ATPase activity of HSP90 and has been evaluated as a potential anticancer drug in clinical trials [22]. Compared with the first-generation HSP90 inhibitor, NVP-AUY922 has lower toxicity and higher therapeutic potential [23]. Studies have reported that inhibiting HSP90 can attenuate acute lung inflammation [24, 25], and that HSP90 is involved in the regulation of inflammatory signal networks [26, 27]; however, it remains unclear whether HSP90 is involved in regulating ferroptosis during radiotherapy. Thus, this study investigates whether NVP-AUY922 inhibits ferroptosis and protects against RILI through the HSP90/CMA pathway in vivo and in vitro.

## Results

### NVP-AUY922 protects against radiation-induced cell injury in BEAS-2B cells

**Fig. 1:**
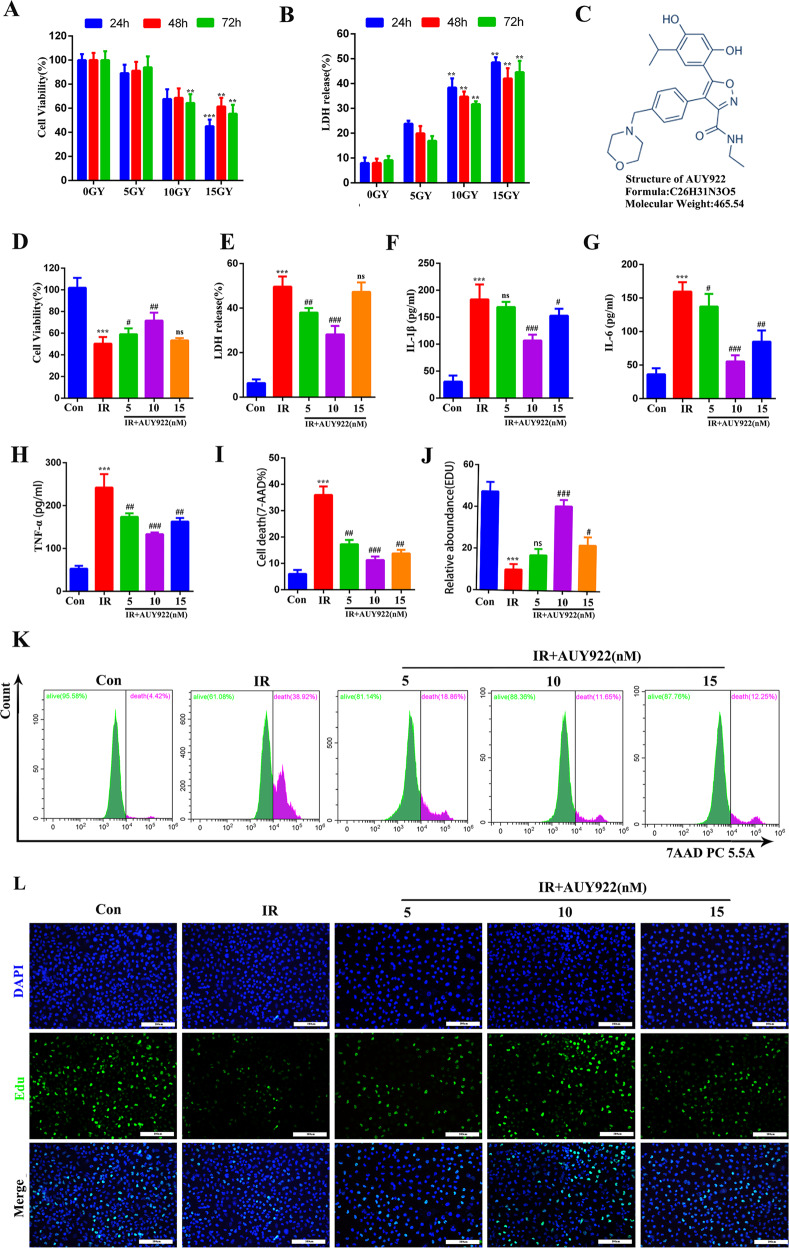
NVP-AUY922 alleviates radiation-induced BEAS-2B cell injury. **A**, **B** Cell viability and LDH release assay was detected at different time points (24, 48, or 72 h) following radiation (0, 5, 10, or 15 Gy). **C** The chemical structure of NVP-AUY922. **D**, **E** Cell viability and LDH release assay in BEAS-2B. **F**–**H** Concentrations of IL-1β (**F**), IL-6 (**G**), and TNF-α (**H**) in cell culture supernatant. **I**, **K** Representative flow cytometric images. **J**, **L** Representative EDU assay fluorescence images. All data are expressed as the mean ± SD. (*n* = 6). ^#/^*P < 0.05, ^##/^**P < 0.01, ^###/^***P < 0.001, ^#^ vs. IR group; * vs. control group.

We established a model of radiation-induced cell injury in BEAS-2B cells. Cell viability was significantly decreased at a radiation dose of 15 Gy at 24 h (Fig. 1A). LDH levels were prominently increased at the same dose and time (Fig. 1B), hence we selected a radiation dose of 15 Gy and time point of 24 h for subsequent cell experiments. Irradiated cells were treated with different concentrations of NVP-AUY922. As shown in Fig. 1D, E, NVP-AUY922 (10 nM) significantly protected the proliferative ability and decreased the LDH release of radiation-treated BEAS-2B cells. Furthermore, the levels of inflammatory factors including TNF-α, IL-6, and IL-1β decreased significantly after 10 nM NVP-AUY922 treatment (Fig. 1F–H). The flow cytometry and EdU staining results showed that 10 nM of NVP-AUY922 had the best protective effect against radiation-induced BEAS-2B cell injury (Fig. 1I–L). These results indicate that the appropriate dose of NVP-AUY922 (10 nM) effectively alleviated radiation-induced BEAS-2B cell injury.

### NVP-AUY922 alleviates RILI

**Fig. 2:**
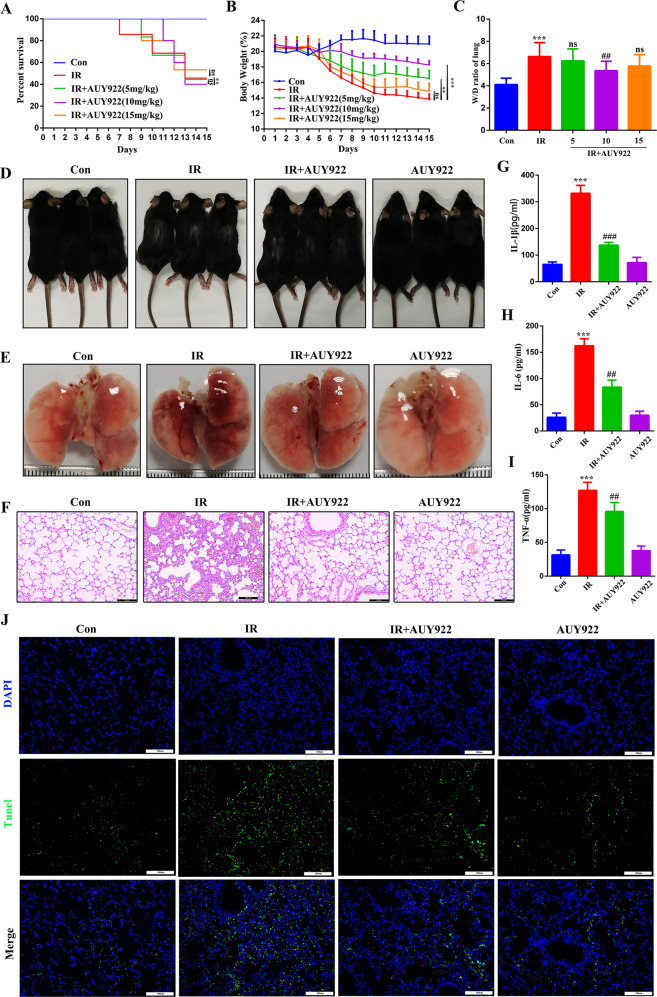
NVP-AUY922 improved survival and lung injury induced by IR in mice. **A** Survival curves. **B** Body Weight. **C** W/D ratio of the lungs. **D** Depilation of mice after radiation. **E** Representative images of lung tissues. **F** Representative HE staining images of the lung tissues. **G**–**I** Concentrations of IL-1β (**G**), IL-6 (**H**), and TNF-α (**I**) in serum. **J** TUNEL staining of lung sections. Mice (*n* = 12) were pre-treated with NVP-AUY922 (5 mg/kg, 10 mg/kg, 15 mg/kg, i.p.) or vehicle (i.p.) 2 h prior to a single radiation dose of 10 Gy (dose rate of 2.0 Gy/min), then continuously administered from day 2 to day 5. Animals were sacrificed on day 16. ^#/^*P < 0.05, ^##/^**P < 0.01, ^###/^***P < 0.001, ^#^ vs. IR group; * vs. control group.

We tested the effect of NVP-AUY922 on the survival and weight of C57BL/6 mice treated with 10 Gy radiation. Mice in the IR group began to die by the 7th day after irradiation, with a survival rate of 41.7% on the 15th day. The survival rates of the NVP-AUY922 (5, 10, and 15 mg/kg) groups reached 58.3%, 75%, and 50%, respectively. Moreover, the survival rate of the NVP-AUY922 (10 mg/kg) group was significantly higher than that of the other groups, with a survival rate of 75% on day 15 (Fig. 2A). Consistently, we observed that 10 mg/kg NVP-AUY922 pre-treatment significantly attenuated radiation-induced loss of body weight (Fig. 2B). The wet-to-dry ratio of the lung reflects the degree of pulmonary edema in the mouse RILI model. Compared to the control group, the wet-to-dry ratio increased significantly in the IR group. Additionally, 10 mg/kg of NVP-AUY922 treatment significantly inhibited the increase in the wet-to-dry ratio caused by radiation (Fig. 2C). Hence, we selected 10 mg/kg of NVP-AUY922 as the appropriate concentration for subsequent animal experiments and found that NVP-AUY922 significantly attenuated radiation-induced depilation and pulmonary hyperemia (Fig. 2D, E). H&E staining revealed radiation-induced pathological changes, including edema, alveolar rupture, and inflammatory cell infiltration (Fig. 2F). NVP-AUY922 treatment significantly ameliorated these pathological changes. Furthermore, NVP-AUY922 pre-treatment significantly inhibited the secretion of IL-1β, IL-6, TNF-α and alleviated radiation-induced cell death (Fig. 2G–J). These results illustrate that NVP-AUY922 reduces lung damage and inflammation caused by radiation.

### Fer-1 inhibition of ferroptosis suppresses RILI

**Fig. 3:**
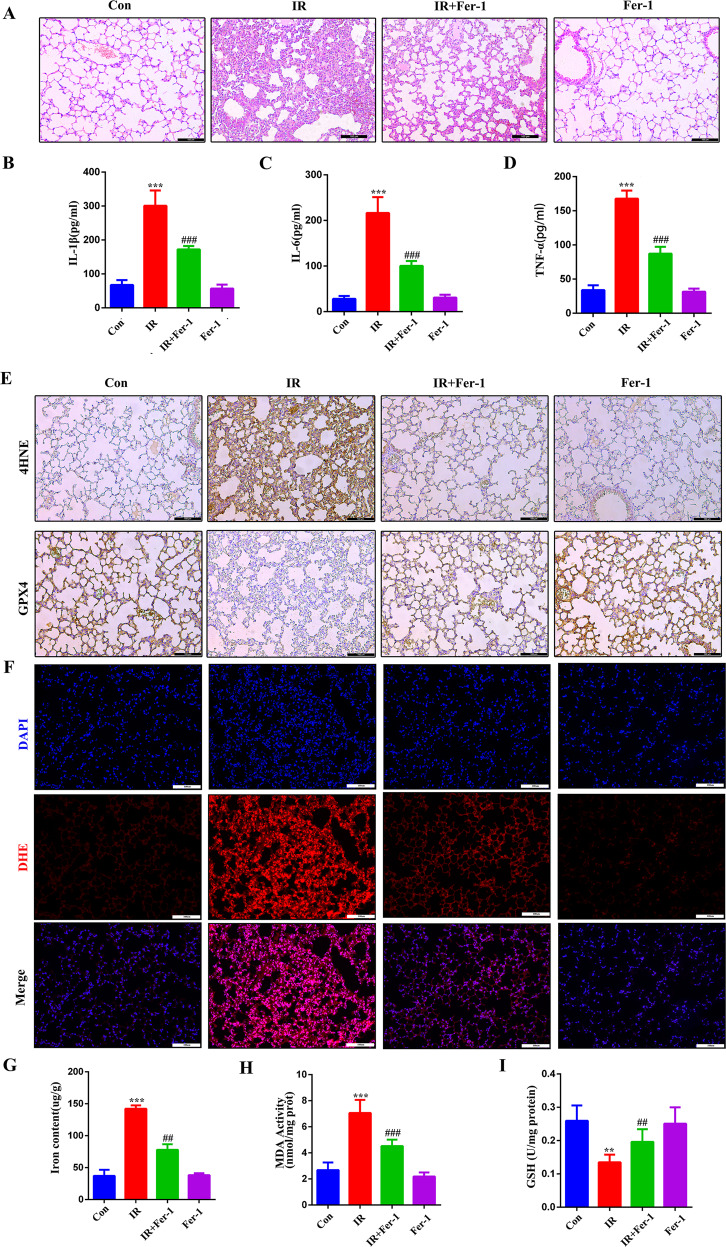
Ferroptosis is involved in radiation-induced lung injury. The mice were treated with ferrostatin-1 (5 mg/kg) or DMSO. **A** H&E staining of lung. **B**–**D** Concentrations of IL-1β (**B**), IL-6 (**C**), and TNF-α (**D**) in the Serum. **E** Representative IHC images of GPX4 and 4HNE in the lung sections. **F** Representative DHE fluorescence images of the mouse lung tissues. **G**–**I** Activities of iron content, MDA, and GSH in the mouse lung tissues. ^#/^*P < 0.05, ^##/^**P < 0.01,^###/^***P < 0.001, ^#^ vs. IR group; * vs. control group.

Radiation-induced accumulation of many lipid peroxides occurs in RILI [28] and is an important factor in ferroptosis [29]. A previous study showed that ferroptosis plays a proinflammatory role [30]. Mice were administered chest radiotherapy at a dose of 10 Gy, and the ferroptosis inhibitor Fer-1 (5 mg/kg) was injected at 2 h before radiotherapy. H&E staining performed to analyze inflammatory infiltration of the lung revealed that mice pre-treated with Fer-1 had dramatically alleviated inflammatory infiltration (Fig. 3A). Moreover, the serum levels of IL-1β, IL-6, and TNF-α were significantly increased in the IR group compared to other group (Fig. 3B–D). In addition, radiation significantly increased the production of excessive lipid peroxide 4-HNE (Fig. 3E), ROS (Fig. 3F), Fe^2+^ (Fig. 3G), and MDA (Fig. 3H) and decreased the levels of GPX4 (Fig. 3E) and GSH (Fig. 3I), which are the characteristic indicators of ferroptosis. In contrast, Fer-1 markedly inhibited radiation-induced changes in these indicators of ferroptosis. These results indicate that ferroptosis is involved in RILI and that Fer-1 can inhibit the lung inflammatory response during RILI in mice.

### NVP-AUY922 suppresses radiation-induced ferroptosis in vitro

**Fig. 4:**
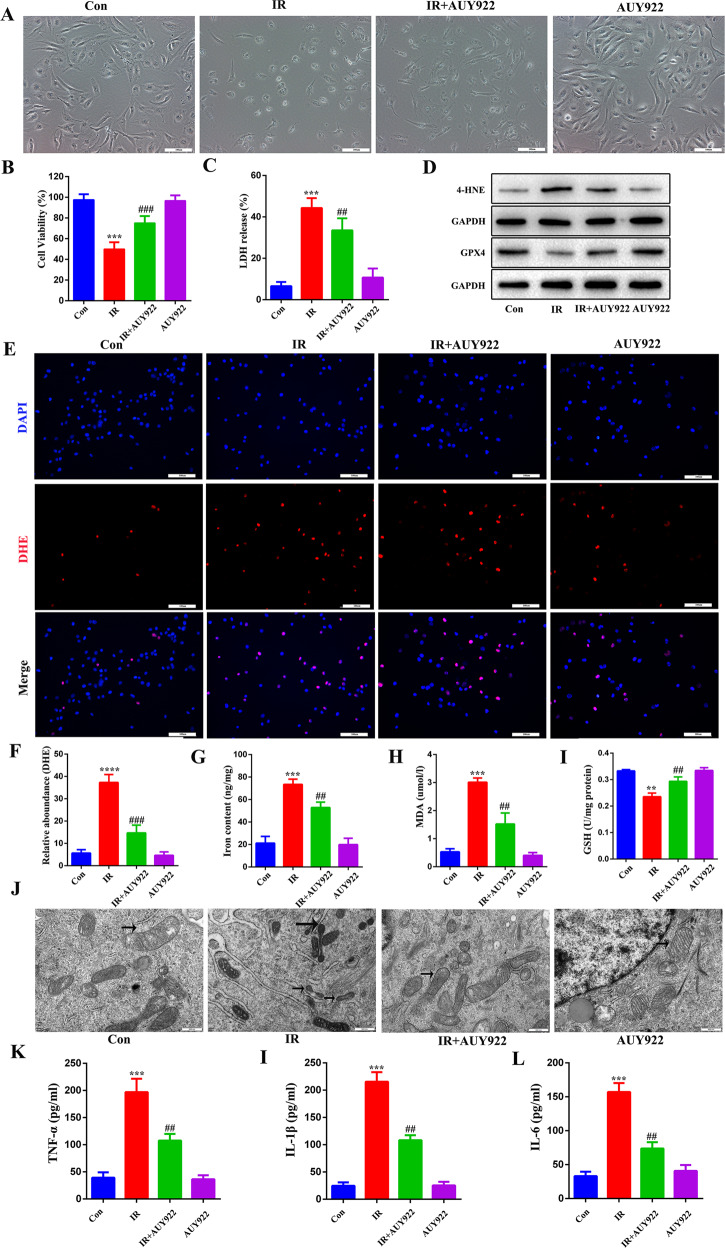
NVP-AUY922 attenuates radiation-induced ferroptosis in BEAS-2B cells. **A** BEAS-2B cell morphology and death were observed. **B**, **C** CCK-8 assays and LDH release assays in BEAS-2B cell. **D** The levels of GPX4 and 4HNE in BEAS-2B cells. **E**, **F** Representative DHE fluorescence images of BEAS-2B cells. **G**–**I** Activities of iron content, MDA, and GSH in cell culture supernatant. **J** Mitochondria with obvious characteristics of ferroptosis were detected by transmission electron microscopy. **K**–**L** Concentrations of TNF-α (**K**), IL-1β (**I**), and IL-6 (**L**) in cell culture supernatant. ^#/^*P < 0.05, ^##/^**P < 0.01, ^###/^***P < 0.001, ^#^ vs. IR group; * vs. control group.

We confirmed the radioprotective role of NVP-AUY922 by cell morphology, cell viability, and LDH release assays (Fig. 4A–C). We also assessed indices of ferroptosis, including increased levels of 4-HNE (Fig. 4D), ROS (Fig. 4E, F), Fe^2+^ (Fig. 4G), and MDA (Fig. 4H) and degradation of GSH (Fig. 4I) and GPX4 (Fig. 4D) in the IR group compared to the control group. As expected, NVP-AUY922 significantly attenuated radiation-induced ferroptosis in BEAS-2B cells. Moreover, transmission electron microscopy was used to detect the morphological changes of mitochondria in BEAS-2B cells, which showed that NVP-AUY922 alleviated radiation-induced mitochondrial damage (Fig. 4J). At the cellular level, NVP-AUY922 significantly reduced radiation-induced inflammatory factors (Fig. 4K–L). Together, these results indicate that NVP-AUY922 inhibits radiation-induced ferroptosis.

### NVP-AUY922 inhibits radiation-induced ferroptosis in vivo

**Fig. 5:**
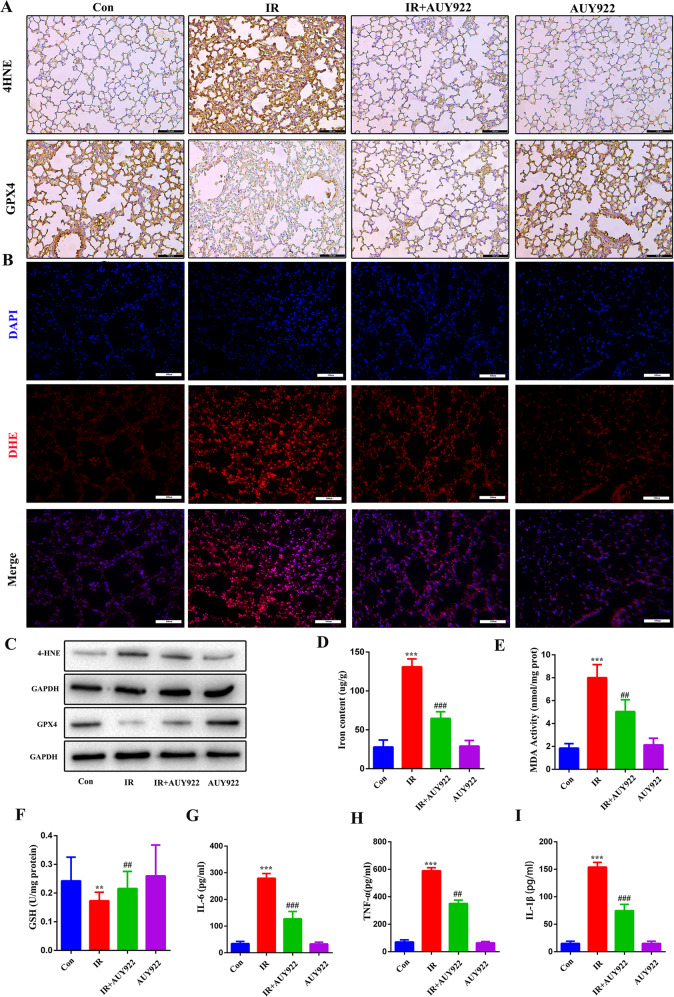
NVP-AUY922 attenuates ferroptosis and inflammation induced by IR. **A** Representative IHC images of GPX4 and 4HNE in the lung sections. **B** Representative DHE fluorescence images of the mouse lung tissues. **C** The levels of 4HNE and GPX4 in lung tissues. **D**–**F** Activities of iron content, MDA, and GSH in the mouse lung tissues. **G**–**I** Concentrations of IL-6 (**G**), TNF-α (**H**), and IL-1β (**I**) in serum. ^#/^*P < 0.05, ^##/^**P < 0.01, ^###/^***P < 0.001, ^#^ vs. IR group; * vs. control group.

The ferroptosis level was evaluated in the lung tissues by detecting the levels of ferroptosis-related proteins using immunohistochemistry and western blotting. The results showed that the level of GPX4 was decreased and 4HNE was increased in the radiation-treated mice, and that NVP-AUY922 pre-treatment reversed this effect (Fig. 5A, C). Other characteristic indices of induced ferroptosis were highly expressed in the lung of radiation-treated mice, including Fe^2+^, MDA, and ROS; as expected, the levels of these components (Fig. 5B–D) were significantly decreased and GSH (Fig. 5F) levels were remarkably increased in the IR + NVP-AUY922 group compared to the IR group. We investigated the levels of inflammatory cytokines that mediate the development of lung injury (Fig. 5G–I), and NVP-AUY922 significantly reduced the expression of these inflammatory mediators. These results indicate that NVP-AUY922 alleviates radiation-induced ferroptosis in mice.

### NVP-AUY922 alleviates radiation-induced ferroptosis by inhibiting the HSP90-mediated CMA pathway

**Fig. 6:**
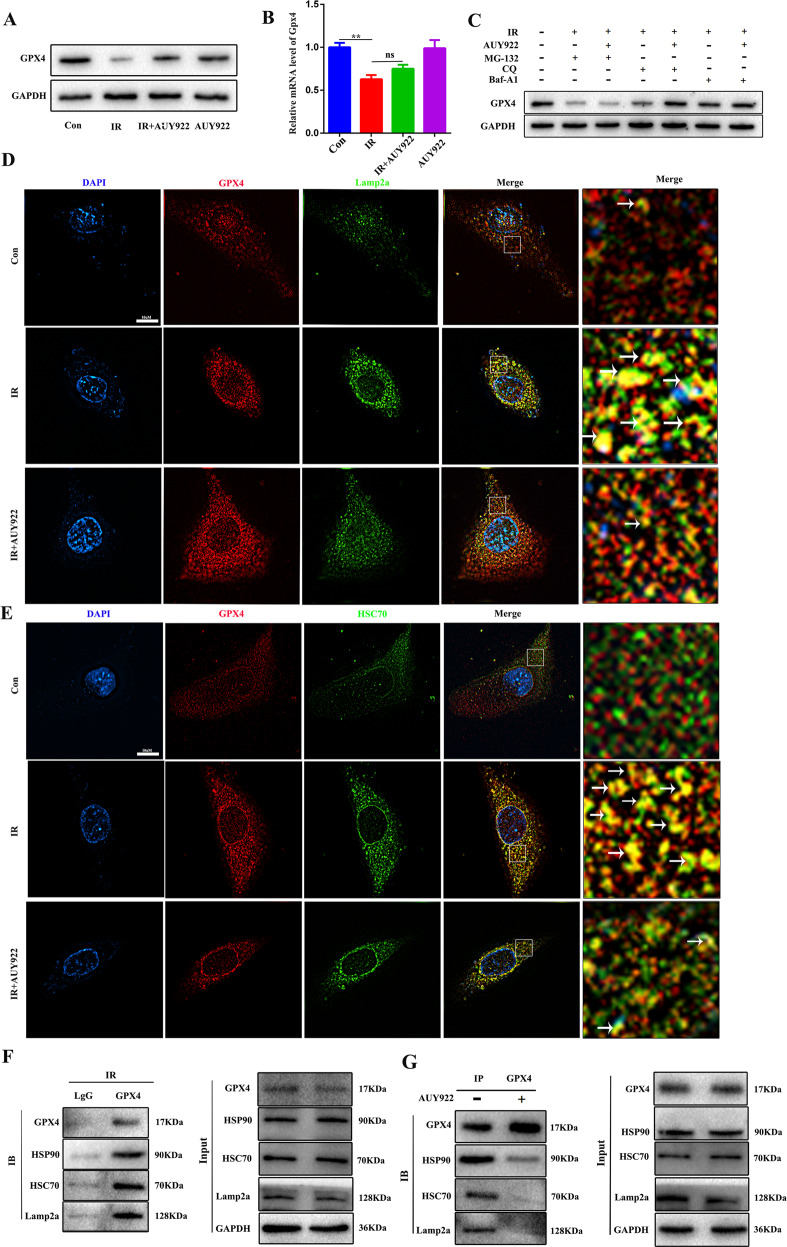
NVP-AUY922 inhibited the interaction between GPX4, HSC70, and LAMP-2A induced by IR. **A** Western blot assay for GPX4. **B** Quantification of GPX4 mRNA levels by qPCR. **C** Western blot using an antibody against GPX4. BEAS-2B cells were pre-treated with MG132 (10 µM), CQ (25 µM), NVP-AUY922 (10 nM) and Baf-A1 (200 nM) 2 h prior to the 15 Gy radiation for 10 min. **D**–**E** Microscopy was performed to detect the changes in GPX4, HSP90, and LAMP-2A. **F** Co-IP assay was performed using an antibody against GPX4 or control IgG and western blotting for GPX4, LAMP-2A, HSC70, and HSP90 was performed. **G** Co-IP assays were performed using an antibody against GPX4 or control IgG followed by western blotting for GPX4, LAMP-2A, HSC70, and HSP90. ^#/^*P < 0.05, ^##/^**P < 0.01, ^###/^***P < 0.001, ^#^ vs. IR group; * vs. control group.

GPX4 is a pivotal protein involved in ferroptosis whose degradation promotes ROS production and irreversible lipid peroxidation, leading to cell death. Our results show that radiation inhibited the expression of GPX4 protein, whereas NVP-AUY922 increased the levels of GPX4 protein following radiation (Fig. 6A). There was no significant difference in *GPX4* mRNA levels between the IR group and IR + NVP-AUY922 treatment group (Fig. 6B), indicating that regulation of GPX4 by NVP-AUY922 occurs at the protein level. To further evaluate the underlying mechanisms, various protein degradation inhibitors, including proteasome and lysosome inhibitors, were used to examine the role of distinct degradation pathways in radiation-related GPX4 level changes. Only the lysosome pathway inhibitor chloroquine or bafilomycin A1, but not the proteasome inhibitor MG-132, rescued the decrease in radiation-induced GPX4 levels (Fig. 6C). These data suggest that the protective effect of NVP-AUY922 on GPX4 depends on the inhibition of lysosomal degradation. NVP-AUY922 is a potent inhibitor of HSP90. Previous studies indicated that activation of ferroptosis leads to increased HSP90-mediated lysosomal delivery and degradation of GPX4 [31, 32]. Therefore, we predicted that NVP-AUY922 participates in the chaperone-mediated lysosomal transport of GPX4. Confocal and co-immunoprecipitation assays suggested that radiation leads to interactions of HSP90, HSC70, GPX4, and LAMP-2A, which are effectively inhibited by NVP-AUY922 (Fig. 6D–G). Therefore, our data support that NVP-AUY922 inhibits the interaction of HSC70, HSP90, LAMP-2A, and GPX4, which mediate lysosomal transport of GPX4, resulting in ferroptosis inhibition.

### Lysosomal inhibitor Baf-A1 alleviates RILI by inhibiting ferroptosis in mice

**Fig. 7:**
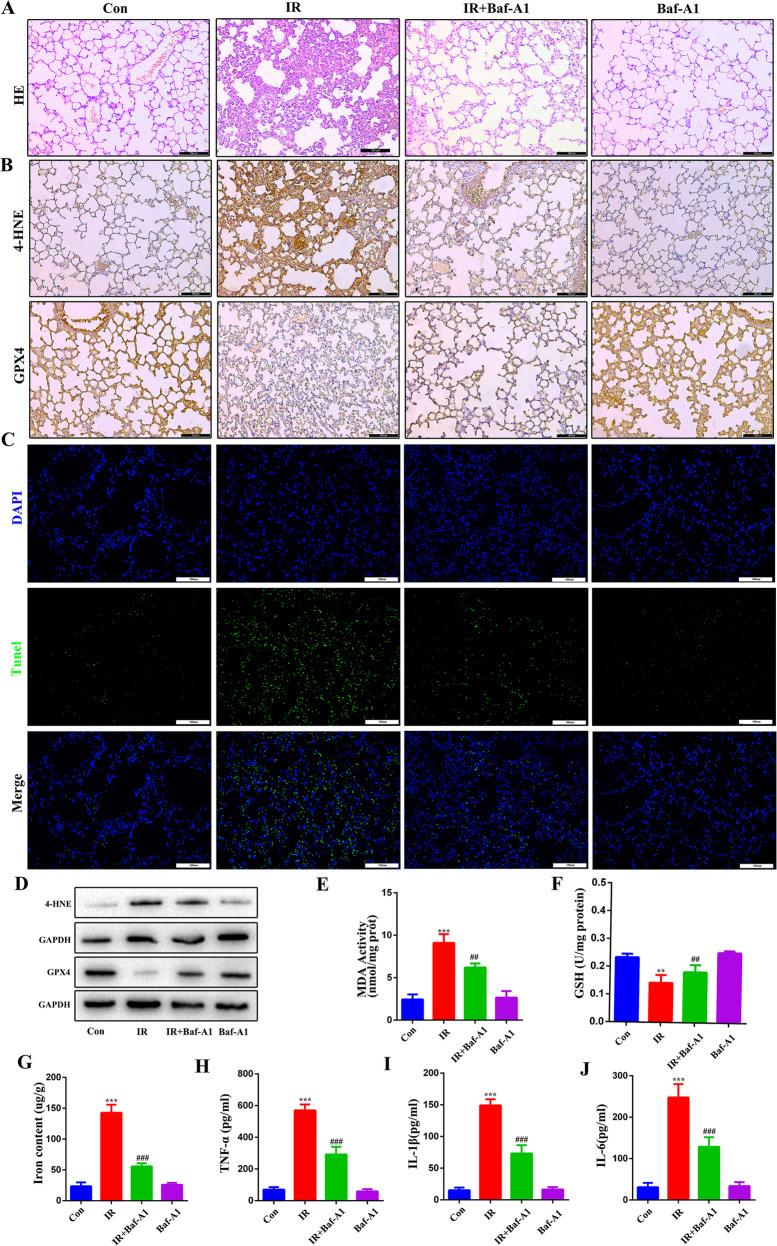
Lysosomal inhibitor of Baf-A1 alleviates radiation-induced ferroptosis in mice. The mice were treated with Baf-A1 (10 µM) or DMSO. **A** H&E staining of lung. **B** Representative IHC images of GPX4 and 4HNE in the lung sections. **C** TUNEL assay of lung sections. **D** The levels of 4HNE and GPX4 in the lung tissues. **E**–**G** Activities of MDA, GSH, and iron content in the mouse lung tissues. **H**–**J** Concentrations of TNF-α (**H**), IL-1β (**I**), and IL-6 (**J**) in serum. ^#/^*P < 0.05, ^##/^**P < 0.01, ^###/^***P < 0.001, ^#^ vs. IR group; * vs. control group.

To further explore whether CMA plays an important role in ferroptosis and RILI, the protective effect of the lysosomal inhibitor Baf-A1 was evaluated in vivo. Immunohistochemistry analysis and western blotting demonstrated that the expression of GPX4 was upregulated in the IR + Baf-A1 group compared to the IR group. Baf-A1 effectively prevented lysosomal degradation of GPX4. Other indicators related to ferroptosis in the lung tissues were significantly improved by Baf-A1 treatment (Fig. 7B, D–G). As shown in Fig. 7 (Fig. 7A, C, H–J), Baf-A1 treatment significantly ameliorated radiation-induced pathological changes, cell death, and secretion of inflammatory factors. Collectively, these data suggest that the lysosomal inhibitor Baf-A1 alleviates RILI by inhibiting ferroptosis and inflammation to some extent.

## Discussion

Lung injury is strongly associated with a poor prognosis in patients treated with radiotherapy [33]. Lung inflammation plays a pivotal role in RILI and can directly or indirectly cause damage to the microvascular endothelium and alveolar epithelium in the lung, which are the primary sources of RILI pathogenesis, and a heavy burden on critically ill patients [34]. Although many candidate molecular targets and drugs have been proposed for the treatment of RILI, the development of therapeutic strategies to improve the results of RILI remains limited; therefore, new drugs or treatments for RILI are warranted. Here, we identified NVP-AUY922 as a potential therapeutic drug for RILI and demonstrated that NVP-AUY922 effectively inhibits HSP90 activity and blocks ferroptosis, thereby alleviating RILI.

NVP-AUY922, a second-generation HSP90 inhibitor used in this study, is one of the most tested HSP90 inhibitors, which can alleviate inflammation [35–37]. However, the therapeutic potential of NVP-AUY922 for RILI remained unclear. In this study, the administration of NVP-AUY922 effectively inhibited radiation-induced pulmonary pathological changes and pulmonary inflammation in vivo and significantly decreased radiation-induced ferroptosis. Many studies have demonstrated that ferroptosis contributes to the progression of RILI [38–40].

Ferroptosis is induced by the inactivation of an essential metabolic process that leads to iron catalysis and lipid- and ROS-mediated cell collapse [41]. In this study, in vivo experiments confirmed that Fer-1 and NVP-AUY922 significantly protected the lung against radiation-induced acute lung damage. Fer-1 and NVP-AUY922 pre-treatment can inhibit the expression and release of inflammatory cytokines and the infiltration of inflammatory cells in lung tissue. In addition, GPX4 was effectively restored by Fer-1 and NVP-AUY922 pre-treatment in radiation-induced cell and animal models. Our results show that ferroptosis mediated inflammation in radiation-treated mice, and NVP-AUY922 may ameliorate radiation-induced lung epithelial cell injury and lung inflammation by inhibiting ferroptosis.

To further investigate the potential mechanism of NVP-AUY922 inhibiting ferroptosis in RILI, we examined the regulatory position and mechanism of GPX4. As an enzyme that is essential for the conversion of toxic lipid hydroperoxides into nontoxic lipid alcohols, GPX4 is the main regulator of ferroptosis [42]. Consumption of GPX4 can lead to overwhelming lipid peroxidation and cell death [43, 37]. The activation of ferroptosis results in an increase in LAMP-2A levels to promote CMA, which, in turn, mediates the degradation of multiple CMA substrates, including GPX4 [44]. CMA is a selective degradation pathway for substrates with KFERQ-like motifs. HSC70 and LAMP-2A are key carriers of CMA pathway molecules; the former is responsible for recognizing specific substrates with KFERQ-like sequences, and the latter is responsible for translocation of target proteins to the lysosome [45].

LAMP-2A recognition of the substrate is considered as a speed-limiting factor in CMA [46]. HSP90 binds to LAMP-2A on the lysosomal membrane and HSP90 regulates CMA mainly by maintaining LAMP-2A stability [47, 48]. Herein, we found that the proteasome inhibitor treatment had no effect on the protein expression level of GPX4, whereas the lysosomal inhibitor Baf-A1 significantly alleviated the degradation of GPX4. Colocalization of GPX4, LAMP-2A, and HSP90 further verified the degradation of GPX4 in the lysosome. Hence, CMA activation mediates ferroptosis.

Our study showed that HSP90 is involved in the occurrence of ferroptosis. Although it is not clear whether HSP90 directly binds to GPX4, we have found that HSP90 participates in ferroptosis by regulating the CMA pathway. Our findings indicate that HSP90 is involved in the pathogenesis of RILI by participating in CMA regulation of GPX4, and the HSP90 inhibitor NVP-AUY922 inhibits radiation-induced ferroptosis and inflammation.

In summary, we found that: (1) NVP-AUY922 relieved radiation-induced BEAS-2B cell damage and RILI; (2) Ferroptosis is involved in radiation-induced damage and ferroptosis may mediate inflammation; (3) NVP-AUY922 may inhibit radiation-induced inflammation and ferroptosis in vivo and in vitro via the HSP90-mediated CMA pathway; and (4) Baf-A1 significantly attenuates radiation-induced inflammation and ferroptosis. These findings suggest that NVP-AUY922 is a promising therapeutic candidate for RILI.

## Materials and methods

### Reagents

Glyceraldehyde 3-phosphate dehydrogenase (60004-1-Ig) and horseradish peroxidase-conjugated secondary antibodies (SA00001-1 and SA00001-2) were purchased from Proteintech (Wuhan, China). Antibodies against Lamp-2a (ab125068), HSP90 (ab32568), GPX4 (ab252833), HSC70(ab51052), and 4-HNE (ab46545) were obtained from Abcam (Cambridge, UK). NVP-AUY922 and ferrostatin-1 (Fer-1) were purchased from Selleck Chemicals (Houston, TX, USA).

### Animal model and experimental design

Female C57BL/6 mice (5–7 weeks, 20 ± 2 g) were obtained from Chengdu Dossy Experimental Animals Co., Ltd. (Sichuan, China). All mice were housed at 25 °C with a 12 h light/dark cycle and maintained in plastic cages with free access to food and water. Mice were randomly assigned to the following four groups (*n* = 12 or 6/ group): Control group, IR group (received radiation), treatment group (injected with NVP-AUY922/Fer-1 and received radiation), and NVP-AUY922 group/Fer-1 group (injected with NVP-AUY922/Fer-1 and no radiation). Mice were anesthetized and administered a single radiation dose of 10 Gy to the thorax. The beam was 6 MV X-ray at a dose rate of 2.0 Gy/min. The radiation field was 2.5 × 2.5 cm. Non-irradiated mice underwent the same procedure but were not exposed to radiation. For the drug-treated groups, NVP-AUY922 was injected intraperitoneally (5, 10, and 15 mg/kg) at 2 h before IR and again daily for 5 days following IR, and the survival rate and weight changes of mice in each group (*n* = 12 per group) were recorded for 15 days. The control group and Fer-1 groups (*n* = 6 per group) were treated with an equivalent volume of dimethyl sulfoxide or Fer-1 (5 mg/kg, intraperitoneal injection), respectively, once daily for seven days (days 1–7). The mice were sacrificed, and samples were collected at 8 days after radiation. All testing and data analysis were conducted in a blinded manner. All experimental protocols were approved by the Laboratory Animal Ethical Committee at Chengdu Medical College.

### Cell culture and radiation treatment

The human normal lung epithelial cell line BEAS-2B was purchased from the Cell Bank of the Chinese Academy of Sciences (Shanghai, China) and cultured in RPMI 1640 medium (Hyclone, Logan, UT, USA) supplemented with 10% (v/v) fetal bovine serum and 5 mg/ml penicillin-streptomycin at 37 °C under 5% CO_2_. The cells were treated with different doses (0, 5, 10, or 15 Gy) of X-ray irradiation at a rate of 2.0 Gy/min. Cell viability and lactate dehydrogenase (LDH) release assays were performed after irradiation for 24, 48, and 72 h. In some experiments, BEAS-2B cells were pre-treated with different concentrations (5, 10, and 15 nM) of NVP-AUY922 for 2 h and exposed to 15 Gy radiation. After 24 h, cell viability, LDH release, and flow cytometry assays were performed.

### EdU assay and TUNEL staining

Cell proliferation was detected using EdU Staining Proliferation Kit (KeyGen, Nanjing, China), and DNA fragmentation was detected using TUNEL Apoptosis Detection Kit (KeyGen). All steps were performed in strict accordance with the corresponding reagent instructions.

### Measurement of glutathione and lipid peroxidation levels

The relative glutathione (GSH) concentration in the lung tissues and cells was assessed using a GSH assay kit (Beyotime Biotechnology), and the level of malondialdehyde (MDA) in the BEAS-2B cells and lung tissues was measured using the Lipid Peroxidation MDA Assay Kit (Beyotime Biotechnology) according to the manufacturer’s instructions.

### Quantitation of ROS

Dihydroethidium (DHE; Molecular Probes, Eugene, OR, USA) staining was used to detect ROS levels in the BEAS-2B cells and lung tissues. The sections were dewaxed and dehydrated using an ethanol gradient and washed with phosphate-buffered saline (PBS); tissue sections or BEAS-2B cells were stained with 5 mmol/l DHE (in PBS) for 20 min at 25 °C. The number of nuclei was assessed using 4′,6-diamidino-2-phenylindole (DAPI) staining. Finally, fluorescence images of the lung tissue sections or BEAS-2B cells were randomly captured at 200× magnification using a fluorescence microscope (DM4000B, Leica, Wetzlar, Germany), and fluorescence intensity was analyzed using ImageJ software (NIH, Bethesda, MD, USA).

### Co-immunoprecipitation assays

The BEAS-2B cells lysates were incubated with the primary antibody with slow rocking overnight at 4 °C. On the second day, protein A + G agarose was added and subjected to slow rocking at 4 °C for 3 h. The samples were centrifuged, and the supernatant was aspirated. The pellet was washed five times with PBS containing 1× phenylmethylsulfonyl fluoride protease inhibitors. The supernatant was aspirated, and the pellet was resuspended in 1× SDS-PAGE loading buffer and incubated in a boiling water bath for 5 min. The samples were then subjected to SDS-PAGE.

### Confocal microscopy

BEAS-2B cells were seeded onto sterile coverslips in specific culture dishes. NVP-AUY922 (10 nM/ml) was added to the wells and incubated for 3 h, followed by 15 Gy radiation treatment 24 h later. Immunofluorescence staining was performed, followed by DAPI counterstaining. A confocal laser scanning microscope was used to collect images.

### Statistical analysis

All experiments were performed independently at least three times. All animals were randomly assigned to experimental groups. Survival was analyzed using log-rank test. Statistical significance among groups was determined using one-way analysis of variance or paired *t*-tests. Statistical significance was set at *P* < 0.05. Statistical analyses were performed using GraphPad Prism 7 software (GraphPad, Inc., La Jolla, CA, USA).

Cell counting kit (CCK-8) assay, LDH release assay, Enzyme-linked immunosorbent assay, Western blotting, Hematoxylin and eosin (H&E) staining and immunohistochemistry, Cell death assay and Real-time PCR analysis please see Supplementary Information.

## Supplementary information

Supplemental Information

## Data Availability

The data that support the findings of this study are available from the corresponding author upon reasonable request.
