# Supplementary material for: NVP-AUY922 alleviates radiation-induced lung injury via inhibition of autophagy-dependent ferroptosis
Source: Cell Death Discov. 2022 Feb 26;8:86. doi: 10.1038/s41420-022-00887-9 (PMC8882174; doi:10.1038/s41420-022-00887-9)

**Supplemental Materials and methods:**

***Cell counting kit (CCK-8) assay and LDH release assay***

BEAS-2B cells in the logarithmic growth phase were inoculated into a 96-well plate at a density of approximately 5 × 10^3^ cells per well and incubated overnight in a cell incubator at 37 °C and 5% CO_2_. The LDH release assay was performed after the corresponding treatment according to the manufacturer’s instructions of the LDH Cytotoxicity Assay Kit (Shanghai, China).

BEAS-2B cells (approximately 5000 cells/well) were seeded into 96-well plates and subjected to the different treatments. After 24, 48, or 72 h, the relative number of viable cells was determined by incubating the cells with the reagents supplied in the CCK-8 kit (Selleck); the optical density of the microplate was measured at 450 nm.

***Enzyme-linked immunosorbent assay***

The cell culture supernatant and mouse serum were collected and centrifuged at 3,000 rpm for 20 min, and the concentrations of IL-1β, IL-6, and TNF-α were detected using enzyme-linked immunosorbent assay kits (Nanjing, China) according to the manufacturer’s instructions. The remaining serum was stored at -80 °C until further analysis.

***Western blotting***

Lung tissues and BEAS-2B cells were lysed in ice-cold RIPA lysis buffer (Beyotime Biotechnology). The protein concentration was measured using a BCA reagent kit (Beyotime Biotechnology); proteins were denatured at 100 °C for 15 min, and total protein samples (40 mg) were separated by 12% sodium dodecyl sulfate-polyacrylamide gel electrophoresis (SDS-PAGE), and transferred onto polyvinylidene difluoride membranes (Millipore, Billerica, MA, USA). The membranes were blocked at 25 °C for 1.5 h and then incubated with primary antibodies at 4 °C overnight. The following primary antibodies were used: Rabbit anti-GPX4 (14432–1-ap, Proteintech, 1:1000), rabbit anti-4HNE (ab31163, Abcam, 1:2000), and rabbit anti-glyceraldehyde 3-phosphate dehydrogenase (KGAA002, KeyGen, 1:10000). Membranes were then stained at 37 °C for 2 h with secondary antibodies conjugated with horseradish peroxidase, and immunoreactive signals were detected by enhanced chemiluminescence (SuperSignal; Pierce, Rockford, IL, USA). Protein signals were detected using a Chemi Doc XRS instrument and Image Lab Software.

***Hematoxylin and eosin (H&E) staining and immunohistochemistry***

The lung tissue was fixed in 4% paraformaldehyde and embedded in paraffin after dehydration. Sections (5-μm-thick) were dewaxed, dehydrated, and washed with PBS; the paraffin sections were stained according to the instructions of the hematoxylin and eosin staining kit (Beijing, China). The samples were then analyzed using an immunohistochemistry detection kit (ZSGB-BIO Technology Co., Ltd., Beijing, China). The remaining samples were subjected to antigen retrieval using a citrate buffer (pH 6.0) and blocked using 5% normal goat serum at 37 °C for 1 h. Next, the samples were incubated with the primary antibody overnight at 4 °C, and then incubated at 25 °C in peroxidase-labeled universal secondary antibody.

***Cell death assay***

For the cell death assay, BEAS-2B cells were collected, stained with 7-aminoactinomycin D (2 μg/ml in PBS, KeyGen) for 30 min, and washed three times. The cells were then analyzed using a flow cytometer (FACSCalibur, BD Biosciences, Franklin Lakes, NJ, USA), and data were collected for analysis.

***Real-time PCR analysis***

Total RNA samples were isolated from BEAS-2B cells using an RNAprep FastPure kit (TSP413, TSINGKE, Shanghai, China), according to the manufacturer’s instructions. First-strand cDNA was synthesized using a reverse transcription kit (TSK302M, TSINGKE). The relative mRNA expression of GPX4 was assayed using a quantitative real-time PCR kit (TSE202, TSINGKE). β-Actin was used as an internal control. The PCR primer sequences for the genes were as follows: β-actin forward: 5ʹ-CCTGGCACCCAGCACAAT-3ʹ and reverse: 5ʹ-GGGCCGGACTCGTCATAC-3ʹ; GPX4-forward: 5ʹ-CCCGATACGCTGAGTGTGGTTTG-3ʹ and reverse: 5ʹ-TCTTCGTTACTCCCTGGCTCCTG-3ʹ.

Original uncropped blots:


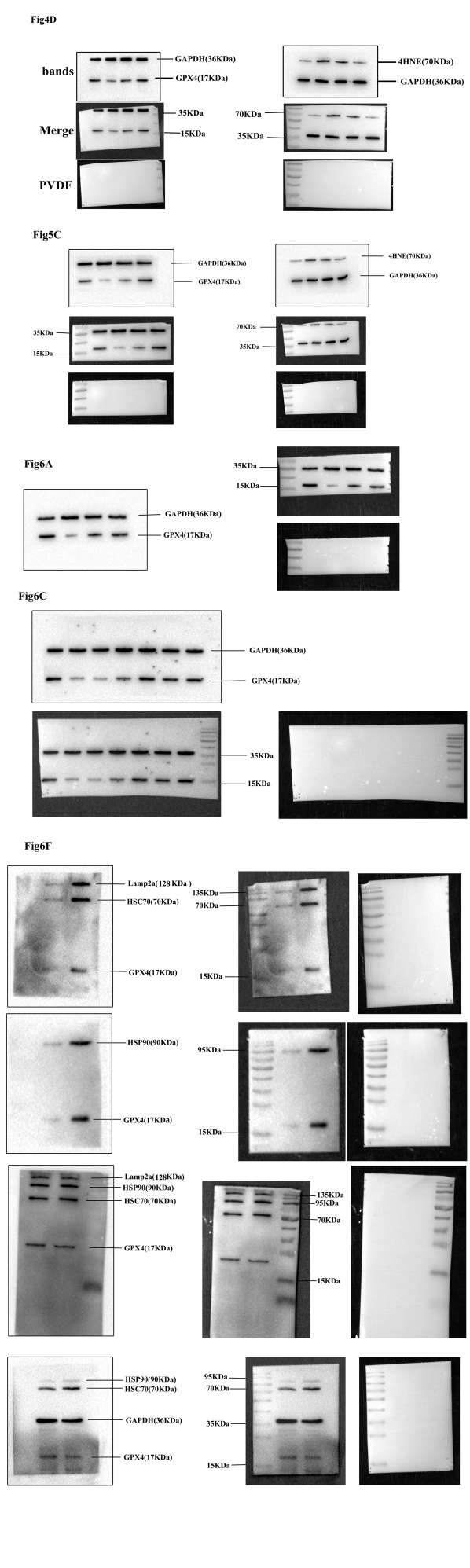


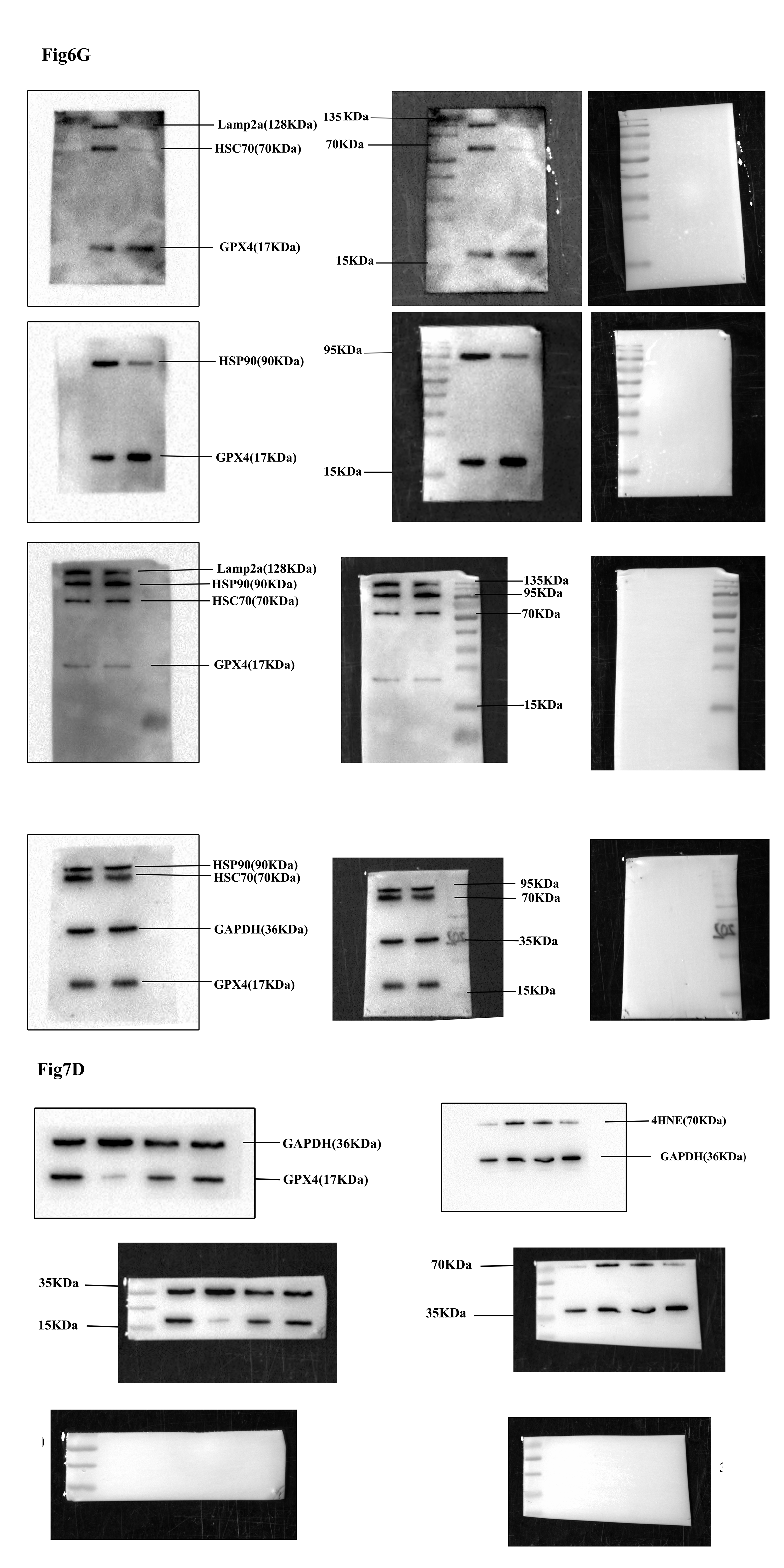

Supplement: Supplementary file 1 — Supplemental Information [file 41420_2022_887_MOESM1_ESM.docx]
